# ‘Asking for help’: a qualitative interview study exploring the experiences of interpersonal counselling (IPC) compared to low-intensity cognitive behavioural therapy (CBT) for women with depression during pregnancy

**DOI:** 10.1186/s12884-021-04247-w

**Published:** 2021-11-12

**Authors:** Jenny Ingram, Debbie Johnson, Heather A. O’Mahen, Roslyn Law, Iryna Culpin, David Kessler, Lucy Beasant, Jonathan Evans

**Affiliations:** 1 Centre for Academic Child Health, Bristol Medical School, University of Bristol, Bristol, UK; 2 Mood disorders Centre, University of Exeter, Exeter, UK; 3 Anna Freud National Centre for Children and Families, London, UK; 4 Centre for Academic Mental Health, Bristol Medical School, University of Bristol, Bristol, BS8 1NU UK

**Keywords:** Interpersonal counselling, Perinatal depression, Qualitative interviews

## Abstract

**Background:**

Treating depression early in pregnancy can improve health outcomes for women and their children. Current low-intensity psychological therapy for perinatal depression is a supported self-help approach informed by cognitive behavioural therapy (CBT) principles. Interpersonal counselling (IPC) may be a more appropriate low-intensity talking therapy for addressing the problems experienced by pregnant women with depression. A randomised feasibility trial (ADAGIO) has compared the acceptability of offering IPC for mild-moderate antenatal depression in routine NHS services compared to low-intensity CBT. This paper reports on a nested qualitative study which explored women’s views and expectations of therapy, experiences of receiving IPC, and Psychological Wellbeing Practitioners (PWPs - junior mental health workers) views of delivering the low-intensity therapy.

**Methods:**

A qualitative study design using in-depth semi-structured interviews and focus groups. Thirty-two pregnant women received talking therapy within the ADAGIO trial; 19 contributed to the interview study from July 2019 to January 2020; 12 who had IPC and seven who had CBT. All six PWPs trained in IPC took part in a focus group or interview. Interviews and focus groups were recorded, transcribed, anonymised, and analysed using thematic methods.

**Results:**

Pregnant women welcomed being asked about their mental health in pregnancy and having the chance to have support in accessing therapy. The IPC approach helped women to identify triggers for depression and explored relationships using strategies such as ‘promoting self-awareness through mood timelines’, ‘identifying their circles of support’, ‘developing communication skills and reciprocity in relationships’, and ‘asking for help’. PWPs compared how IPC differed from their prior experiences of delivering low-intensity CBT. They reported that IPC included a useful additional emotional component which was relevant to the perinatal period.

**Conclusions:**

Identifying and treating depression in pregnancy is important for the future health of both mother and child. Low-intensity perinatal-specific talking therapies delivered by psychological wellbeing practitioners in routine NHS primary care services in England are acceptable to pregnant women with mild-moderate depression. The strategies used in IPC to manage depression, including identifying triggers for low mood, and communicating the need for help, may be particularly appropriate for the perinatal period.

**Trial registration:**

ISRCTN 11513120. 02/05/2019.

**Supplementary Information:**

The online version contains supplementary material available at 10.1186/s12884-021-04247-w.

## Background

Early detection of depression during pregnancy is important because depression can adversely affect birth outcomes and neonatal health, and if untreated can persist postnatally. Treating depression early in pregnancy can improve mother-infant attachment, and the cognitive, emotional, and behavioural outcomes for children [1]. UK guidelines recommend that midwives screen for antenatal depression at the woman’s first midwife appointment using depression screening questions and regularly ask women about their current mental health during pregnancy [2]. Although qualitative studies have shown that both midwives and women regard screening for antenatal depression as acceptable and important [3], screening alone is not enough and referral to appropriate treatment is important.

There is limited evidence for the effectiveness of non-pharmacological psychological interventions for antenatal depression [4] and which treatments might be most appropriate. This is especially true for low-intensity therapies [5] and is important in healthcare systems that have adopted stepped-care approaches where the first offer is typically a low-intensity therapy.

Current talking therapy for mild to moderate depression during pregnancy as provided by Improving Access to Psychological Therapies (IAPT), a national stepped-care, primary healthcare psychological service in England, is a supported self-help approach informed by cognitive behavioural therapy (CBT) principles [2].

However, only offering low-intensity CBT for pregnant women may be problematic, because some studies have reported difficulties enrolling and/or retaining pregnant or postpartum women in CBT [6–8]. This may be because the CBT approach is not relevant or addressing the specific problems of the perinatal period [9]. O’Mahen’s qualitative study highlighted that women in the perinatal period struggled with internalization of “motherhood myths,” self-sacrifice, and managing social support during this period [9]. CBT focusses on people’s thoughts and behaviours, and this may miss difficulties with emotion around transitions, difficulties around communication and support (highlighted as important by women), and issues with complicated grief [9]. Thus, a significant number of women may miss the opportunity to engage with treatments that are meaningful or hold face validity for them. Consequently, several recent studies have modified CBT to improve feasibility and acceptability among pregnant women [10–13].

Another promising talking therapy is Interpersonal Counselling (IPC), which is a low-intensity treatment derived from Interpersonal Psychotherapy (IPT) [14]. IPC may be more appropriate for addressing the problems that depressed women experience during pregnancy and postnatally. It helps individuals to develop useful strategies to manage depression in an interpersonal context and can involve a partner if appropriate. It focuses on strategies to manage changes in role, conflict, isolation and loss (such as miscarriage, still-birth, previous loss of would-be grandparents), and the impact of these on relationships. However, there are limited data on the effectiveness and acceptability of IPC perinatally and no studies of IPC in pregnancy in the UK. A small feasibility study of IPC for antenatal depression in the US amongst low-income mothers indicated high satisfaction with IPC and some improvement in mood [15]. There are currently no studies comparing IPC with low-intensity CBT in the perinatal period.

A randomised feasibility trial (ADAGIO) comparing the acceptability of offering IPC for antenatal depression in routine NHS primary care services in England compared to low-intensity CBT has recently been completed [16, 17]. The ADAGIO trial was successful in recruiting pregnant women with mild-moderate depression. Treatment adherence was high (over 70% of women completed their IPC course to the satisfaction of the Psychological Wellbeing Practitioners (PWPs) delivering the therapy); women reported IPC was acceptable, and supervisors reported high treatment fidelity in IPC PWPs [17]. PWPs are junior mental health workers trained to assess and support people with common mental health problems (principally anxiety disorders and depression) in the management of their recovery.

A nested qualitative study explored the views of participating pregnant women about their expectations of therapy and experiences of receiving IPC, and the views of the PWPs who delivered the low-intensity therapy. The aim of this paper is to understand the views of women and PWPs about these talking therapies in pregnancy, with a particular focus on IPC.

## Methods

### Setting

The ADAGIO feasibility trial was undertaken in two geographical sites in England. Twelve existing PWPs working for the IAPT services were recruited and six were trained to deliver IPC (three at each site), the other six received a short top-up in CBT using perinatal specific guided self-help. Differences between the boundaries of IAPT services and midwifery services in each location, meant that the population from which to identify potentially eligible women for the study was smaller (by approximately 50%) at one site.

### Participants

The ADAGIO study recruited 52 pregnant women (12–26 weeks’ gestation) with mild to moderate depression from January to September 2019. Participants were screened for depression using Edinburgh Depression Scale (EPDS) [18] score 10 or above, and ICD-10 mild or moderate depression determined by the Clinical Interview Schedule Revised (CIS-R) [19], a structured diagnostic computerised psychiatric interview. Randomisation was carried out remotely by the Bristol Clinical Trials Unit stratified by recruiting centre and minimised by parity (with random block sizes). Of those recruited, 42 participants provided follow-up data and 32 received their allocated talking therapy (either IPC or CBT).

Women who received therapy (either IPC or CBT) were purposively sampled to approach for an interview to achieve a maximum variation sample in terms of study arm, maternal age and parity, and study site. Women were approached by telephone and email for interview by DJ (qualitative researcher) after they had completed their therapy and telephone interviews took place between July 2019 and January 2020. DJ also took detailed notes of several shorter telephone conversations with those who had declined or dropped out of therapy. DJ had met two-thirds of the women at recruitment to the trial and they knew that she was a member of the study team. Five of the PWPs who were trained in IPC were involved in online focus group discussions (led by JI and DJ – both experienced qualitative researchers) and one who could not attend was interviewed by DJ. Interview topic guides were informed by the research literature, team discussions and input from our Patient Advisory Groups. Interviews took between 25 and 75 min (median 50 mins) and the two focus groups were 60 and 70 min long.

### Analysis

Thematic data analysis was carried out by trained qualitative researchers (DJ, LB, JI) who have extensive experience of qualitative research and evaluation of health care services, from psychology, health services research and midwifery backgrounds. Interviews and focus groups were audio-recorded, transcribed verbatim by a professional transcription service and anonymised. Analysis of the data was an ongoing and iterative process using NVivo 11 software to organise and code the transcripts [QSR International Pty Ltd]. Transcripts were initially coded by one qualitative researcher (DJ). Codes and themes were developed and discussed with the lead qualitative researcher (JI) at regular intervals during data collection and analysis to achieve consensus. Six interview transcripts were also read and coded by an independent qualitative researcher (LB) to compare and discuss the coding framework [20]. Interviews continued until data saturation was achieved, in that no new themes were arising from the data. All analytical decisions were shared and discussed by the qualitative research group using a consensus process to agree the final coding and thematic framework.

The study received North of Scotland Research Ethics Committee (REC) approval on October 29th 2018 and Health Research Authority approval on November 14th 2018.

## Results

A total of 19 women who had received therapy contributed to the qualitative study; 16 were interviewed (11 who had IPC; five had CBT) and three had telephone conversations (one IPC, two CBT) giving opinions from 12 who had IPC and seven who had CBT. All women who were approached for interview agreed, but two were not available for a while due to imminent induction of their babies. The women in the study had a mean age of 32.6 years (range 25–42); nine were expecting their first baby and 10 were expecting their second or subsequent baby. All six PWPs trained in IPC at both sites were interviewed. Quotes from women and PWPs are presented: women are identified by site and therapy arm; and PWPs by site.

### 1.Themes from all therapy interviews

Overall, pregnant women were positive about receiving therapy through the ADAGIO study, welcomed being asked about their mental health in pregnancy, and having the chance to have support in accessing therapy. For some the study offered the first opportunity to acknowledge and explore their low mood and it was the only time they were offered help and treatment.

“Just very grateful for the opportunity that I have had. I would never have thought of therapy … I just don’t think I had the insight to do so, and my GP didn’t offer anything like that either.” (#1021,site A)

Themes from all the interviews are reported initially to describe the overall expectations of therapy as ‘engagement with antenatal depression therapy’, ‘tools for life’ and ‘PWP insights’.

#### Engagement with therapy for antenatal depression

Some women were unsure about how helpful the treatments might be, but a willingness to engage with their sessions enabled them to get the most out of either therapy, even if they were initially doubtful.

“I wasn’t really sure how … I would respond to the interpersonal counselling, but I was interested to see whether it would help. I knew that it was good to be going and talking to them when I’m addressing my feelings anyway, I think having that structure can be helpful when you are struggling”. (#2009, site B)

Women’s perspectives often changed throughout their course of treatment so that by completion, they were able to reflect upon the benefit of persisting with exercises and acknowledge positive changes that had occurred.

#### Tools for life

Women found both treatments focused on practical issues, and when they engaged with these they were pleased with their therapy. Both therapies offered ‘tools for life’ and women appreciated being given things to do or handouts that they could refer to later.

“Accepting help was a big issue, … .. so she would say why don’t you just try and accept help for this and see how it goes, and then she would say things like your homework this week is to think about more ways of self-care, those kinds of things”. (#1018, site A, IPC)

“The coping mechanisms make sense, and they were explained well, and they do work, I do believe they work if you practice them, so I did feel I got help.” (#1025, site A, CBT)

Women in both therapy groups talked about being encouraged to do ‘homework’ between sessions to put strategies into practise that had been identified in their sessions. In CBT these appeared to be drawn from an existing set of exercises.

“I think once I really tried to do the short exercises and homework, for want of a better word, I found them more and more useful as I went through.” (#2002, site B, CBT)

Whereas IPC’s approach provided a framework for identifying and making goals which helped women start focusing on specific issues that could be affecting their mood and that they ‘wanted to tackle’.

“It wasn’t you must do it, but it was much more let’s think of an action out of this if that makes sense? .,.. well now you have said that, what do you think you could do or what do you think could be different, if that makes sense? It was like someone was listening a bit more and actually trying to think of a solution.” (#1021, site A, IPC)

#### PWP insights

PWPs commented that the task-orientated low-intensity CBT approach is more therapist-led and to them it lacked the emotional component of IPC, which they felt was more responsive to women’s emotional experiences. IPC practitioners welcomed this new element in their sessions as they believed it enabled a more holistic approach to women’s therapy.

“I felt more 'with' the client I guess, I understood their emotional perspective. I understood how they were feeling in the room a little bit more, potentially because we were talking about emotions, and asking “how does that make you feel right now?”, whereas with CBT we are very much focused on how do we use this technique, how can we use it at home. So, I did feel maybe slightly more emotional connection with the clients.” (PWP focus group, site A)

### 2. Interpersonal counselling interview themes

This more emotion-focussed approach of IPC helped pregnant women to identify triggers for low mood. These were facilitated through exploring interpersonal specific depression triggers, using exercises such as a timeline of depression, relationship mapping and circles of support. Women spoke of IPC in terms of working collaboratively with their practitioner to develop solutions to their issues often saying “we did this or that”. Importantly they did not feel they were being told what to do, rather, they reported that they were helped to find strategies that would work for them, with their PWP sometimes suggesting ideas:

“It definitely wasn’t a case of her saying you need to do this, you need to do that, you very much get there together.” (#1018, site A, IPC)

“I was worried that once we stopped seeing each other that actually I would become quite down and depressed after the baby is born, so we looked into that and we looked into the support group as to who could help the emotional side, who I know I can speak to, and she told me that I could go back to them at any point as well.” (#2005, site B, IPC)

Focussing on views of women who received IPC (*n* = 12) and the PWPs (*n* = 6) who delivered it, the themes generated included ‘promoting self-awareness through mood timelines’, ‘circles of support’, ‘communication skills and relationships’, and ‘asking for help’. PWPs also compared delivering both therapies.

#### Promoting self-awareness though mood timelines

Women reported that the IPC strategies supported them to increase their self-awareness, identify their support networks, and learn to ask for help. IPC practitioners initiated the process by helping them to make mood timelines*,* which women felt helped them to recognise their triggers for low mood.

“She did this activity, it was a timeline of looking at what’s been going on in my life, when I’ve been experiencing low mood, anxiety, depression, and what perhaps happened before that and what happened after, and that was a really useful activity to do actually, to map it all out. That was really great, and just her listening and validating how I was feeling was quite comforting.” (#1029, site A, IPC)

#### Circles of support

Identifying their support networks of people, who they could call upon, was an important step towards women starting to ask for help. They were encouraged to identify their ‘circle of support’ by creating a diagram with the practitioner which most found to be helpful:

“I remember doing one exercise where you were encouraged to draw on all the people of support in your life, so to really look at who you would talk to, like your friends, your family, people at work and that sort of thing. So you had to draw a physical diagram, [ … ] equally I think that the goal of that type of therapy is that you are using all those supports and you realise it’s okay to talk to those people about things that was going on.” (#1021, site A, IPC)

“ … having the circle, so knowing who is in the support group and [name] reflected on that actually, and there was a chart that she gave me where I could write who it was, my relationship with them, and what good they bring me, and can I rely on them for emotional and physical support, that was really helpful to go through, to know who I had and who I could rely on.” (#2005, site B, IPC)

#### Communication skills and relationships

Women were encouraged to work specifically on developing their communication skills, identifying problem areas in their relationships with others, recognising the reciprocal nature of communication and helping them to try out different approaches with the aim of improving the way they manage such interactions. Both women and PWPs were able to see the benefit of working on these issues, which resulted in very positive changes for some.

“..expressing what my needs are to people, friends, partner, wherever, work, making sure it’s clear what my needs are rather than just assuming people are going to know, because they don’t. So I have tried to apply that for sure and I would say that’s been helpful.” (#1034, site A, IPC)

“Probably all the stuff about how you communicate and the words you use rather than … and how that might make the other person feel or be defensive, and that was all quite positive.” (#1014, site A, IPC)

“There was freedom and a different focus, still depression, but there was focus on relationship that’s not really the main thing in CBT, and I think a lot of clients that I worked with found that helpful and having space to talk about things a bit more freely it seemed like it was helpful.” (PWP focus group, site A).

#### Asking for help

Reaching out to others was difficult for many women as it involved acknowledging their low mood, possibly for the first time, and then opening up to other people to ask for help. Most women admitted finding it difficult to seek or accept help and it seemed that working through this in IPC could be powerful in enabling positive changes in women’s behaviour.

“We’d worked really closely on reaching out to other people and letting people know how I was feeling, and a lot of it was asking for help which I used to be terrible at. [ … .] I went from not wanting to speak to anyone to being very open about it, and it gave me a huge amount of confidence to speak to my health visitor about it, [ … ..] I definitely felt more empowered to speak out about how I was feeling for sure.” (#1006, site A, IPC)

### 3. Comparison of IPC with CBT

Most PWPs delivering IPC were surprised at how different it was from low-intensity CBT, and initially felt under-prepared to deliver the new therapy. However, they gained confidence with each participant and ultimately reported that they enjoyed the opportunity to try out a different approach which they felt was more woman-centred and highly pertinent to the perinatal period.

“I was expecting something completely different, so there were some elements of the structure that reminded me of CBT, but I would say IPC allowed more freedom, more space for building a therapeutic relationship that maybe CBT at times lacks, especially if you focus quite rigidly on everything.” (PWP focus group, site A)

“It was definitely nice to be able to experience a bit more freedom when talking with people and bringing emotions much more into the room. I think CBT doesn’t ignore those things, but it doesn’t actively talk about them, and so it’s quite nice to be able to check in every single week with actual emotions and to give someone that space.” (PWP focus group, site A)

“ … I can see a role in perinatal/antenatal period, with the communication, just some of those really simple things we were doing with the communication it can make a real difference at that low intensity level.” (PWP focus group, site B)

The relationship between the PWP and client was different between the two therapies, requiring PWPs to adapt to be more responsive to issues women wanted to talk about in IPC. Most welcomed the opportunity to learn the different approach IPC demanded of them and enjoyed being able to encourage women to talk about their feelings.

## Discussion

This study has highlighted that it is possible to deliver low-intensity interpersonal counselling for depression during pregnancy in large community settings. Women and practitioners liked IPC and found it to be very relevant to the perinatal context. They particularly highlighted that it helped to identify triggers for depression and communicating the need for help. They valued the exploration of relationships using strategies such as a mood timeline, relationship mapping and circles of support. PWPs welcomed the opportunity to learn a more emotion-focussed approach to treating pregnant women. This qualitative study was part of a feasibility trial of IPC compared to CBT and the interviews also aimed at assessing the acceptability of the new therapy. The findings will inform the delivery of the trial processes in a larger trial which will focus on effectiveness and cost-effectiveness.

Other studies exploring views of perinatal mental healthcare within IAPT have shown that women reported positive experiences of receiving support from IAPT for perinatal mental health difficulties. IAPT services are encouraged to prioritise perinatal women so that they can be offered timely help with their depression. However in some studies, both women and therapists have highlighted issues relating to barriers to access and a need to tailor therapy to the perinatal context [21].

Finding that CBT provided by low-intensity practitioners is not always relevant to perinatal depression led O’Mahen [9] and others to produce modified tailored training packages to address the perinatal-specific concerns relating to self, motherhood, and interpersonal domains of CBT. These concerns, which notably centred around women’s interpersonal skills and problems, rippled out to affect their negative thoughts and behaviours as well as their resilience and efficacy behaviours. IPC, which targets interpersonal domains, may be ideally appropriate for the concerns which depressed pregnant women express.

Previous evaluations of IPC have focussed on efficacy rather than qualitative views of acceptability. However, one qualitative study has shown that it is likely to be an effective and acceptable treatment for young people with primarily depressive symptoms seen in local authority non-specialist mental health services [22]. Participants described specific advantages of IPC over standard counselling, including practical help, the use of goals, psychoeducation and integrating a self-rated questionnaire into treatment [22]. In our qualitative study, women also highlighted the benefits of identifying specific depression triggers, using exercises such as a timeline of depression, relationship mapping and circles of support. Another small US trial using brief-IPT compared to treatment as usual for perinatal depression showed that it was acceptable to low-income women and helpful for improving depressive symptoms and social support. However, there was relatively low session attendance in that trial, which limited the interpretation of the study results [15]. In our study over 70% of women completed their IPC course to the satisfaction of the PWP delivering the therapy.

Strengths of our qualitative study are being able to include almost 60% of the pregnant women in the trial, who received talking therapy, through interviews and detailed phone conversations, and 40% of those who did not receive therapy. The junior mental health practitioners (PWPs) also provided valuable insights into delivering IPC compared to their usual low-intensity CBT sessions.

Limitations include not being able to talk to the women experiencing depression during pregnancy who did not choose to enter the trial or who were lost to the study along the way.

## Conclusions

Identifying and treating depression in pregnancy is important for the future health of both mother and child. IPC delivered by psychological wellbeing practitioners in routine NHS primary care services in England is a relevant and acceptable psychological therapy for pregnant women with mild-moderate depression. The strategies used in IPC to manage depression, including identifying triggers for low mood, and communicating the need for help, may be particularly appropriate for the perinatal period.

## Supplementary Information

**Additional file 1.**

## Abbreviations

CBT: Cognitive behaviour therapy
IPC: Interpersonal counselling
IAPT: Improving Access to Psychological Therapies
PWP: Psychological Wellbeing Practitioners
