# Supplementary material for: ‘Asking for help’: a qualitative interview study exploring the experiences of interpersonal counselling (IPC) compared to low-intensity cognitive behavioural therapy (CBT) for women with depression during pregnancy
Source: BMC Pregnancy Childbirth. 2021 Nov 12;21:765. doi: 10.1186/s12884-021-04247-w (PMC8588677; doi:10.1186/s12884-021-04247-w)
Supplement: Supplementary file 1 — Additional file 1. [file 12884_2021_4247_MOESM1_ESM.docx]

**‘Asking for help’: A qualitative interview study exploring the experiences of Interpersonal Counselling (IPC) compared to low-intensity cognitive behavioural therapy (CBT) for women with depression during pregnancy.**

**Topic guides for qualitative interviews.**

These are guides only. Interviewer will use further prompts and probes in response to participants’ responses. Interviewer to use own discretion to omit questions or alter wording as appropriate during interviews.

Interviews will take up to 60 minutes. All will be transcribed verbatim and anonymised before analysis. No one will be identified in any published report.

1. **Women in the intervention (IPC) and CBT arms.**

Patients who agree to be interviewed will be contacted by telephone and interviewed on the phone or face-to-face.

For telephone interviews: Confirmation of consent to take part and for interview to be recorded will be recorded verbally.

Face-to-face interviews will complete the consent form.

**Topics to be covered:**

- Reflections on the recruitment process
  - Information given by midwife and researchers
  - Randomisation
- Motivation for joining the study
  - From midwife, partner, others
- Views on the talking therapy that they received
  - Making appointments
  - Relationship with ‘psychological wellbeing practitioner’
  - Involving partner
  - Sessions content and usefulness
- Acceptability of the outcome measures completed
  - Burden to patients, numbers of questions etc.

1. **Partners of those receiving IPC or CBT: interview topics.**

Partners who agree to be interviewed will be contacted by telephone and interviewed on the phone or face-to-face.

For telephone interviews: Confirmation of consent to take part and for interview to be recorded will be recorded verbally.

Face-to-face interviews will complete the consent form.

**Topics to be covered:**

- Reflections on the recruitment process
  - Information given by midwife and researchers
  - Randomisation
- Views on the talking therapy that they received
  - Making appointments
  - Relationship with counsellor
  - Sessions content and usefulness
  - Benefit of the sessions to partner

1. **Study decliners will be interviewed by phone only.**

These will be brief telephone interviews lasting 10 - 15 minutes unless women wish to talk for longer.

- Reasons for dropping out/declining
- Ways in which continuing participation could have been supported

1. **Staff Interview – practitioners in IPC arm, supervisors and midwives.**

Staff who agree to be interviewed will be contacted and interviewed by phone. The IPC practitioners from both sites will be invited to attend focus groups to discuss their experiences.

Topics will include:

- Reflections on the recruitment process
- Usual care for women screened at risk of depression
- Encouraging women to take part
- Views on the talking therapies
  - Strengths
  - Weaknesses
